# A general-purpose Monte Carlo particle transport code based on inverse transform sampling for radiotherapy dose calculation

**DOI:** 10.1038/s41598-020-66844-7

**Published:** 2020-06-17

**Authors:** Ying Liang, Wazir Muhammad, Gregory R. Hart, Bradley J. Nartowt, Zhe J. Chen, James B. Yu, Kenneth B. Roberts, James S. Duncan, Jun Deng

**Affiliations:** 1 Department of Therapeutic Radiology, School of Medicine, Yale University, 15 York Street, New Haven, CT 06510-3221 USA; 2 Department of Biomedical Engineering, School of Engineering & Applied Science, Yale University, 300 Cedar Street, New Haven, CT 06520-8042 USA

**Keywords:** Radiotherapy, Biomedical engineering, Theoretical particle physics

## Abstract

The Monte Carlo (MC) method is widely used to solve various problems in radiotherapy. There has been an impetus to accelerate MC simulation on GPUs whereas thread divergence remains a major issue for MC codes based on acceptance-rejection sampling. Inverse transform sampling has the potential to eliminate thread divergence but it is only implemented for photon transport. Here, we report a MC package Particle Transport in Media (PTM) to demonstrate the implementation of coupled photon-electron transport simulation using inverse transform sampling. Rayleigh scattering, Compton scattering, photo-electric effect and pair production are considered in an analogous manner for photon transport. Electron transport is simulated in a class II condensed history scheme, i.e., catastrophic inelastic scattering and Bremsstrahlung events are simulated explicitly while subthreshold interactions are subject to grouping. A random-hinge electron step correction algorithm and a modified PRESTA boundary crossing algorithm are employed to improve simulation accuracy. Benchmark studies against both EGSnrc simulations and experimental measurements are performed for various beams, phantoms and geometries. Gamma indices of the dose distributions are better than 99.6% for all the tested scenarios under the 2%/2 mm criteria. These results demonstrate the successful implementation of inverse transform sampling in coupled photon-electron transport simulation.

## Introduction

The Monte Carlo (MC) method is a numerical solution to a problem based on random statistical trials. The method represents a probabilistic model of object-environment or object-object(s) interactions^1^. Through this method many quantities of interest can be predicted, even those that are hard to measure^2^. The ability to solve difficult mathematical problems, the computational speed, and the memory capacity of modern digital computers are the main drive of the extremely rapid development and application of the MC method in almost all major scientific areas^3–6^. This method offers reasonable solutions to many problems in the fields of therapeutic and diagnostic radiology dosimetry (e.g., calculation of fundamental dosimetric quantities, radiation doses, and simulations of radiotherapy treatment planning) and radiation protection (e.g., personal radiation doses and radiation shielding calculations). As MC methods can accurately model the physical processes which occur during the particle transport in media^7,8^, its applications in these fields have increased exponentially since the 1970s^9–13^. Because of its stochastic nature, simulation of a large number of particle histories is required to achieve a desired statistical accuracy. The early applications of MC methods were restricted to simple geometries due to the relatively limited computational capabilities of early computers. These limitations were overcome with the availability of modern computing power and simulations of complex geometries (e.g., medical linear accelerator treatment heads, radiation detectors, and patient phantom based on CT images) became possible^13^. A number of general purpose MC based simulation codes are currently available for the radiation transport, e.g., MCNP, Geant4, EGSnrc, and PENELOPE^10,14–18^.

The general-purpose MC radiation transport packages have a long history of applications in radiotherapy^3,9,19,20^, where accurate patient-specific dose calculation plays a crucial role^21–25^. Efforts have been made to improve simulation efficiency by launching general-purpose MC radiation transport packages on graphics processing units (GPUs), including GPUMCD^19^, gDPM^20,26,27^, and GMC^28^. However, thread divergence remains a major issue since those GPU codes are adapted from the CUP counterparts that use the acceptance-rejection method. The number of iterations required to sample a physical interaction from the probability density function (PDF) obeys the Poisson distribution. In a GPU execution, the threads finish the sampling procedure earlier have to wait all the other threads finish sampling before they can move collectively to the next instruction. The inverse transform method has the potential to reduce thread divergence because it only requires one iteration to sample an interaction from the inverse cumulative distribution function (CDF). The application of this method has been demonstrated for photon transport^29^. However, it has not been implemented in a coupled photon-electron transport scenario.

The main goal of this study is to develop a general-purpose Monte Carlo package named Particle Transport in Media (PTM) to demonstrate the full implementation of the inverse transform sampling technique in coupled photon-electron transport simulation on CPU. Tabulated data libraries of the inverse CDFs are created for all the physical models related to photon and electron transport. Sampling algorithm is implemented using the inverse transform sampling scheme, i.e. a random number between 0 and 1 is generated to represent the cumulative probability and a linear interpolation is applied to obtain the physical quantity from the inverse CDF data library. Benchmark studies against both EGSnrc simulations and experimental measurements are performed for various beams, phantoms and geometries. The developed PTM package can be used to calculate radiation doses received by patients in diagnostic imaging, treatment simulation, verification and delivery. This work demonstrated the feasibility of inverse transform sampling in coupled photon-electron transport simulation on CPU and paved the way for its implementation on GPU.

## Methods

### Scheme of photon transport simulation

A simulation history (photon) is initiated by sampling the photon source parameters such as energy, position and direction. The photon is transported along a straight line to the first interaction site where an interaction is forced to occur. Tabulated values of total cross-section for each interaction type of photon (i.e., coherent scattering, Compton scattering, photoelectric effect and pair production) and the first three photoionization subshell cross-sections (K, L1, and L3) against energy for all listed neutral atoms in the Periodic Table computed by Cullen^30^ were used. The path length is selected based on the total cross-section^30^ of the material through which the photon is being transported. A photon may go through various interactions including coherent/elastic (Rayleigh) scattering, inelastic (Compton) scattering, photoelectric effect, and pair/triplet production. The probability of each interaction is proportional to the corresponding cross-sections^30^ for the material at the interaction site. The photon is then transported to the next interaction site and is forced to interact with the medium. This process continues until the photon history is terminated by a preset photon energy cutoff, Russian Roulette^18,31,32^ (a technique to randomly terminate the photon history) or the interaction site being outside the system/geometry boundary. The energy transfer to the material at each interaction site is stored and thus is available for calculation of different quantities (e.g., flux and dose). During the transport process, secondary particles (i.e., photons, electrons, and positrons) are also produced from the above stated interactions. They are stored in respective secondary particle banks and the secondary photons are tracked in the same way as the primary photon after terminating the primary history. The treatment of each interaction type for a photon is described in the following sections.

### Coherent/elastic scattering

In the coherent scattering of a photon by a bound atomic electron, the photon changes its direction only with negligible energy transfer to the primary target atom. After scattering, the photon trajectory is deflected by an angle θ in the interval (0, π), and symmetrically about the initial axis of propagation at an azimuthal angle φ in the interval (0, 2π). As there is negligible change in the photon energy after coherent scattering, one only needs to sample the angular deflection of the photon.

Several MC codes based on the rejection technique were previously developed for the angular distribution of coherently scattered photons^1,2,7,9,12,13,16,20,21,33,34^. The azimuthally-symmetric differential cross-section (DCS) for the coherent scattering of the un-polarized photons to polar angle θ can be expressed as follow^10,14,35,36^

1 $$\frac{{\rm{d}}{\sigma }_{{\rm{coh}}}({\rm{Z}},{\rm{E}},\theta )}{d\theta }=\frac{{{\rm{r}}}_{{\rm{e}}}^{2}}{2}(1+{\cos }^{2}{\rm{\theta }})2\pi \,\sin \,{\rm{\theta }}\,{{\rm{F}}}^{2}({\rm{Z}},{\rm{E}},\theta ),$$

where Z is the target-atom’s atomic number, E is the energy of the photon, *r*_*e*_ is the classical Thomson radius of the electron, and the scattering atomic form factor^10,14,35,36^ is,

$${{\rm{F}}}^{2}({\rm{Z}},{\rm{E}},\theta )={({f}_{0}({\rm{Z}},\theta )+f{\prime} ({\rm{Z}},{\rm{E}}))}^{2}+{f}^{{\prime\prime} 2}({\rm{Z}},{\rm{E}}).$$

Here, *f*_0_(Z,*θ*) represents the non-relativistic/relativistic form factors while *f*′(Z,E) and *f*″(Z,E) respectively are the real and the imaginary parts of the complex scattering amplitude of the anomalous scattering factors^36^. Tabulated values of *f*_0_(Z,*θ*) against momentum transfer *q* as well as *f*′(Z,E) and *f*″(Z,E) against photon energy for all listed neutral atoms in the Periodic Table are available in literature^37–40^. Assuming, *f*′(Z,E) = *f*″(Z,E) = 0, when angular distribution of the scattered photon are considered to be *f*′(Z,E) and *f*″(Z,E) independent^10^.

The probability of a photon scattered coherently into the polar angle interval *dθ* around *θ* is given by

2 $$p(\theta )d\theta =\frac{d{\sigma }_{coh}(Z,E,\theta )}{{\sigma }_{coh}({\rm{Z}},E)}=\frac{{r}_{{\rm{e}}}^{2}}{2}\frac{(1+co{s}^{2}\theta ){{\rm{F}}}^{2}({\rm{Z}},{\rm{E}},\theta )2\pi sin\theta d\theta }{{\sigma }_{coh}({\rm{Z}},E)},$$

where, σ_*coh*_ is the total coherent cross section^3,15^.

For MC purposes, let us introduce *μ* = cos*θ* and *α* = *E*/(*m*_*e*_*c*^2^) where *m*_*e*_ is the electron rest mass, use the trigonometric relations to favor instance of the half-angle *θ*/2 (i.e. sin^2^(*θ*/2) = (1−cos*θ*)/2), and favor instance of the momentum transfer *q* using *q* = sin(*θ*/2)/*λ*. The following is the result

3 $${q}^{2}={(k\alpha )}^{2}(1-\mu ),0\le {q}^{2}\le {\bar{q}}^{2},$$

where, $k={m}_{{\rm{e}}}c/(\sqrt{2}h)=29.1445{\AA }^{-1}$, *h* is Planck’s constant, and ${\bar{q}}^{2}$ corresponds to the maximum momentum transfer^10,14,35,36^.

Solving (3) for μ yields *μ* = 1 − [*q*^2^/(*kα*)^2^] and the derivative *dμ*/(*dq*^2^) = −1/(*kα*)^2^. The scattering probability in Eq. (2) can be rewritten in terms of the squared momentum transfer q^2^ instead of the polar angle θ

4 $$p({q}^{2})d{q}^{2}=\frac{d{\sigma }_{coh}(Z,\alpha ,\mu )}{{\sigma }_{coh}(Z,\alpha )}|\frac{d\mu }{d{q}^{2}}|d{q}^{2}=\frac{d{\sigma }_{coh}(Z,\alpha ,\mu )}{{\sigma }_{coh}(Z,\alpha )}|-1/{(k\alpha )}^{2}|d{q}^{2}.$$

The coherent DCS Eq. (1) is similarly written in terms of q rather than θ as,

5 $${{\rm{\sigma }}}_{{\rm{coh}}}({\rm{Z}},{\rm{\alpha }},{\rm{\mu }}){\rm{d}}{\rm{\mu }}={{\rm{\pi }}{\rm{r}}}_{{\rm{e}}}^{2}(1+{{\rm{\mu }}}^{2}){{\rm{F}}}_{0}^{2}({\rm{Z}},{\rm{q}}){\rm{d}}{\rm{\mu }}.$$

By combining the probability (4) and the DCS (5), the scattering probability is related to the coherent cross section by eventual integration over the space of scalar squared momentum transfer

6 $$p({q}^{2})d{q}^{2}=\frac{{{\rm{\pi }}{\rm{r}}}_{{\rm{e}}}^{2}}{{(k\alpha )}^{2}{\sigma }_{coh}(Z,\alpha )}(1+{{\rm{\mu }}}^{2}){F}_{0}^{2}({\rm{Z}},{\rm{q}})d{q}^{2}.$$

A coherent scattering *μ* map as a function of ξ and E is generated based on Eq. (6) for inverse sampling. Specifically, *μ* ∈ [−1, 1] and log(*E*(*MeV*)) ∈ [−6.91, 6.42] are evenly divided into 100 and 250 grids. For a given grid point (*μ*, *E*), the coherent scattering probability is calculated using Eqs. (3) and (6). Repeat this for all the combinations of *μ* and *E*, we obtain a coherent scattering PDF *p*(*μ*, *E*). By integrating *p*(*μ*, *E*) from −1 to *μ*, we arrive at the CDF *ξ* = *P*(*μ*, *E*). The inverse CDF, i.e. the coherent scattering *μ* map, *u* = *P*^−1^(*ξ*, *E*) is then numerically determined. Upon the simulation of a coherent scattering event for a photon with energy E, the scattering angle cosine is simply determined by picking a random number *ξ* between (0, 1) and a subsequent linear interpolation of the *μ* map. The azimuthal angle φ is uniformly distributed within interval (0, 2π).

### Incoherent (Compton) scattering

Incoherent scattering is the major mode of photon interaction with matter for photons with energies used for therapeutic radiation. In this interaction, the collision is between a photon and a nearly-free electron inside the medium. After a collision, a fraction of the photon energy is transferred to the colliding electron and the direction of the photon is changed. This type of scattering is normally referred to as incoherent scattering due to the independent behavior of the electron which prevents any interference effects. While simulating the event, the objective is to sample both the energy Eʹ and *θ* (i.e., angle of deflection from the line of flight) of the scatted photon. The scattered photon is further transported with new direction and energy, Eʹ (lower than initial energy). The energy loss of the photon (i.e., E − Eʹ) at the point of collision produces a Compton recoil electron that is stored in the secondary electron bank for further transport^32^. The differential scattering cross-section of a photon scattered from free electron (generally known as Compton scattering) is given below^31^

7 $$\sigma (Z,\alpha ,\mu )d\mu =S(Z,q)K(\alpha ,\mu )d\mu ,$$

where *S*(*Z*, *q*) is an incoherent scattering function modifying the Klein-Nishina cross-section^31,32^

8 $$K(\alpha ,\mu )d\mu =\pi {r}_{{\rm{e}}}^{2}{\left(\frac{\alpha {\prime} }{\alpha }\right)}^{2}\left(\frac{\alpha {\prime} }{\alpha }+\frac{\alpha }{\alpha {\prime} }+{\mu }^{2}-1\right)d\mu ,$$

where *α* and *α*′ are photon energies in units of the rest mass energy of the electron (i.e., 0.511 MeV) before and after scattering, respectively. The probability density function (PDF) of *μ* is^15,31,32,41^

9 $${p}_{i}(\mu )=\frac{S(Z,q)K(\alpha ,\mu )}{{\sigma }_{i}(Z,\alpha )}={C}_{1}\frac{S(Z,q)K(\alpha ,\mu )}{S(Z,\bar{q}){\sigma }_{k}(\alpha )}.$$

Here, *σ*_*i*_(Z, *α*) and *σ*_*k*_(*α*)^15,31,41^ are the microscopic incoherent scattering cross-section and the integrated Klein-Nishina cross-section, respectively, C_1_ = 1.651035 is a normalization constant^32^, and $\mathop{q}\limits^{-}$ is the maximum momentum transfer for given incident-photon energy *α*. Based on Eq. (9), an incoherent scattering *μ* map as a function of ξ and E is generated following the same procedure as described in the coherent scattering section. The azimuthal angle φ is uniformly distributed within the interval (0, 2π).

The scattered photon energy, *α*′ is calculated from the sampled *μ* which also accounts for scattering off of a bound electron^31^. Due to the incident momentum of the electron, there is a broadening of the scattered photon energy spectrum. The effect is known as Doppler broadening. The scattered energy *α*′of a Doppler-broadened photon is calculated by selecting an orbital shell, sampling the projected momentum from the Hartree-Fock Compton profile, *J*(*p*_*z*_), and calculating the scattered photon energy, *α*′, from^32^

10 $${p}_{z}=-137\frac{\alpha -\alpha {\prime} -\alpha \alpha {\prime} (1-\mu )}{{({\alpha }^{2}-{\alpha }^{{\prime} 2}-2\alpha \alpha {\prime} \mu )}^{1/2}}.$$

Tabulated *J*(*p*_*z*_), values as a function of *p*_*z*_ for the elements are published by Biggs *et al*.^42^. The relation between the incoherent scattering function *S*(*Z*, *q*) and the Hartree-Fock Compton profile *J*(*p*_*z*_) is^32^

11 $$S(Z,q)=\sum _{k}{\int }_{-\infty }^{{p}_{z}^{max}}{J}_{k}({p}_{z},{\rm{Z}}){\rm{d}}{p}_{z},$$

where k represents a particular electron subshell and ${p}_{z}^{max}$ is the maximum momentum transfer calculated using E′ = E − E_binding_.

### Photoelectric absorption

The photoelectric effect is the absorption of the incident photon (energy E) with the consequent emission of several fluorescent photons and the ejection of an orbital electron of binding energy E_binding_. As a result of this process, an electron with kinetic energy of E − E_binding_ along with 0, 1, or 2 fluorescent photons are emitted^32^. In our MC dose engine, there are two ways of describing fluorescence photon emission for the case of photoelectric absorption. (1) By analog simulation: all the detailed physics following photoelectric absorption are simulated based on their physical probabilities. (2) By adopting a particular biasing scheme: a single absorption event can generate only one fluorescence photon. In the first method, either 0, 1, or 2 fluorescence photons are emitted as a result of a single absorption event. The probability of absorption by the i^th^ subshell (i.e., K, L1, L2, L3, etc.) is^31^

12 $${p}_{i}=\frac{{\sigma }_{i}(E)}{{\sigma }^{pe}(E)},$$

where *σ*_*i*_(*E*)^15,31,41^ is the photoelectric absorption cross-section of the i^th^ subshell and *σ*^pe^(*E*)^15,31,41^ is the total photoelectric absorption cross-section. There are three possible transitions from the electron shell vacancy. (1) By fluorescence (or radiative) transition, where the inner shell vacancy is filled with an electron from outer shells and the energy difference of the two shells converts to a fluorescence photon. (2) By Coster-Kronig transition, which is a radiation-less transition between intra-shells. For example, an L1 vacancy may be transmitted to an L3 vacancy without a photon release with its yield defined as *F*_*L*1,*L*3_. (3) By Auger transition. Even though it is now considered a single step physical transition, Auger transition has been described in two steps: (1) inner vacancy is transmitted to an outer shell generating a photon, and (2) the photon kicks an electron out of its shell leaving two or more vacancies.

### Pair production

To simulate a pair production event in the nuclear field, the photon history is terminated with the creation of an electron-positron pair. The excess photon energy (E − 2m_e_c^2^) is converted to kinetic energy and is assumed to randomly split among the electron and positron. This is not exact but its influence on the result is negligible due to the low probability of pair production. The directions of the electron and positron are calculated according to its kinetic energy using equation (2.1.21) in the EGSnrc manual^16^. The electron and positron are stored in the secondary electron/positron bank for subsequent transport. The triplet production process is relatively less probable and is simply treated as a pair production event. The threshold for pair production is 2*m*_*e*_*c*^2^(1 + (*m*_*e*_/*M*)) ≅ 1.022MeV, where M is the mass of the nucleus.

### Scheme of electron transport simulation

Unlike photons, analog simulation of electron transport is prohibitively time consuming because a typical radiotherapy-relevant electron will undergo ~10^5^ ionizations and excitations before the primary and all secondary electrons come to a stop in the medium. To overcome this difficulty, a class II condensed history method outlined by Berger^43^ was employed. In this method, Bremsstrahlung radiation and inelastic scattering events that lead to energy transfer above a certain threshold (catastrophic or hard interactions) are simulated explicitly. Photons and electrons produced in these processes are stored in a secondary particle bank for subsequent simulation. Subthreshold radiative or inelastic scattering events, as well as elastic scatterings are “condensed” in a single electron step delimited by two subsequent catastrophic interactions. The cumulative effects of these condensed events are accounted for by calculating an energy loss based on restricted stopping power and sampling a direction change according to a multiple-scattering angular distribution at the end of the step.

### Catastrophic interactions

Bremsstrahlung processes and inelastic scatterings that lead to energy transfer greater than a predefined threshold are simulated in an analog manner. Bremsstrahlung, or “braking radiation”, is electromagnetic radiation produced by the deceleration of charged particle in the electric field of atomic electrons or the nucleus. A portion of the kinetic energy of the charged particle is given off as a new photon to satisfy energy conservation. The generalized Bremsstrahlung DCS of an electron impinging on a neutral atom can be expressed as

13 $$\frac{d{\sigma }_{B}(T,Z)}{dk}=\frac{{Z}^{2}}{{\beta }^{2}k}f(k,T,Z),$$

where β is the velocity of the incoming electron in units of speed of light, k is the fraction of the electron kinetic energy radiated, T is the kinetic energy of the incident electron, Z is the atomic number of the target atom, and *f*(*k*, *T*, *Z*) is the total scaled Bremsstrahlung energy-weighted spectrum. A comprehensive set of *f*(*k*, *T*, *Z*) data is tabulated by Seltzer and Berger^44^. For energies and elements commonly encountered in the radiotherapy realm, f is a weak function of T and Z. Sempau *et al*.^45^ proposed that it could be roughly represented by a linear function f(k) = a(1 − bk). Coefficients a and b are from a linear fit of the tabulated data. After applying the linear approximation, the total macroscopic cross section of catastrophic Bremsstrahlung is easily obtained via integration of dσ_B_/dk from k_B_ to 1

14 $${\varSigma }_{B}=\frac{an{Z}^{2}}{{\beta }^{2}}\left(\mathrm{ln}\,\frac{1}{{k}_{B}}-b(1-{k}_{B})\right),$$

where n is the atom number density, k_B_ = E_B_/T with E_B_ being the threshold for Bremsstrahlung emission, and T being the kinetic energy of the incident electron. E_B_ is set to 10 keV by default.

The proportion of the emitted photon energy to the electron kinetic energy k is sampled from the inverse cumulative density function (CDF) corresponding to the normalized Bremsstrahlung DCS. The kinetic energy of the electron after Bremsstrahlung is reduced to (1 − k)T accordingly but its direction in motion is assumed to remain unchanged. The polar angle θ of the photon relative to the initial direction of electron is approximated^46^ as its mean value m_e_/(T + m_e_) while the azimuthal angle φ is sampled uniformly between 0 and 2π.

Electron-electron inelastic scatterings resulting in energy losses that are large compared to the binding energy of the atom are described by the Møller cross section^47^

15 $$\frac{d{\sigma }_{M}(T)}{dW}=\frac{2\pi {r}_{e}^{2}{m}_{e}{c}^{2}Z}{{\beta }^{2}{W}^{2}}\left[1+\frac{{W}^{2}}{{(T-W)}^{2}}+\frac{{\tau }^{2}}{{(\tau +1)}^{2}}{\left(\frac{W}{T}\right)}^{2}-\frac{2\tau +1}{{(\tau +1)}^{2}}\frac{W}{T-W}\right],$$

where r_e_ is the classical electron radius, m_e_ is the electron rest mass, Z is the atomic number of the target atom, T is the kinetic energy of the incident electron, β is the electron velocity in units of light, τ = T/m_e_ is the electron kinetic energy in units of m_e_, and W is the kinetic energy of the knock-on electron. Since the two electrons are indistinguishable and the one with lower energy after collision is regarded as the scattered electron, the upper limit value of W is T/2. By integrating the Møller formula (15) from W = E_M_ (inelastic scattering threshold) to W = T/2, we arrive at the total macroscopic Møller cross section

16 $${\sum }_{M}=\frac{2\pi {r}_{e}^{2}{m}_{e}{c}^{2}nZ}{{\beta }^{2}T}[\frac{1-2{\varepsilon }_{M}}{{\varepsilon }_{M}(1-{\varepsilon }_{M})}+\frac{{\tau }^{2}}{{(\tau +1)}^{2}}(\frac{1}{2}-{\varepsilon }_{M})-\frac{2\tau +1}{{(\tau +1)}^{2}}\,\mathrm{ln}(\frac{1-{\varepsilon }_{M}}{{\varepsilon }_{M}})],$$

where n is the atomic number density and ε_M_ = E_M_/T. By default, we use an E_M_ of 200 keV. The total cross section for a compound is obtained via a linear combination of the cross sections of the component elements.

The kinetic energy W of the knock-on electron is sampled from an inverse CDF based on the normalized Møller cross section described in Eq. (15). Since energy transfer is large compared to the binding energy of the atom, an approximation is made that the incident electron inherits the rest of the kinetic energy T-W after collision. The cosines of the polar angle *θ*_1,2_ of both the primary and the secondary electron are given each by

17 $$\cos \,{\theta }_{1,2}=\sqrt{\frac{T{\prime} }{T}\frac{T+2{m}_{e}}{T{\prime} +2{m}_{e}}},$$

where T is the initial kinetic energy of the incoming electron and Tʹ is the kinetic energy of either of the electrons after Møller scattering. The azimuthal angle φ_1_ of the primary electron is sampled uniformly between 0 and 2π while the azimuthal angle of the secondary electron is opposite, i.e. φ_2_ = φ_1_ + π.

### Restricted stopping power

Energy losses due to sub-threshold Bremsstrahlung and inelastic scatterings are classified as restricted stopping power. Restricted Bremsstrahlung stopping power L_B_ imparted to an electron of speed *β* and kinetic energy T by an element of number density n, atomic number Z, and Bremsstrahlung emission threshold E_B_ is

18 $${L}_{B}=\frac{an{Z}^{2}{E}_{B}}{{\beta }^{2}}\left(1-\frac{b{E}_{B}}{2T}\right),$$

The stopping power L_B_ is calculated based on the Bremsstrahlung DCS described in the previous section. Symbols used in this expression have the same meaning as those in Eqs. (13) and (14).

Calculation of restricted inelastic scattering stopping power is not as straightforward as that of Bremsstrahlung because the Møller cross section Eq. (15) is only valid in the regime of collisional energy-transfer much larger than the binding energy of the target atom. For low energy transfer events, the theory of Bethe^34^, in which an explicit summation of the entire excitation spectrum is carried out, should be used instead. By combining Bethe’s theory with the Møller formula, one finds the following formulation for restricted inelastic scattering stopping power *L*_*c*_ imparted to an electron of speed *β* and kinetic energy T = *τm*_*e*_, by an element of number density *n*, atomic number Z, and inelastic scattering threshold *ε*_*M*_,

$${L}_{c}=\frac{2\pi {r}_{e}^{2}{m}_{e}{c}^{2}nZ}{{\beta }^{2}}\left[\mathrm{ln}\,\frac{2(\tau +2){T}^{2}}{{I}^{2}}+F(\tau )-\delta \right],$$

19 $$F(\tau )=-1-{\beta }^{2}+\,\mathrm{ln}[{\varepsilon }_{M}(1-{\varepsilon }_{M})]+\frac{1}{1-{\varepsilon }_{M}}+(1-{\beta }^{2})\left[\frac{{\tau }^{2}{\varepsilon }_{M}^{2}}{2}+(2\tau +1)\mathrm{ln}(1-{\varepsilon }_{M})\right],$$

where I is the target atom’s mean ionization energy and δ is the density correction that accounts for the reduction of collision energy-transfer due to polarization of the bulk medium. Data for the δ of 100 elements and some common materials are taken from the ESTAR database^48^. It should be noted that F(τ) is different for electrons and positrons. In our code, this difference is ignored and positrons are processed in the same way as electrons during flight. This approximation is justified by the relatively low probability of pair production compared to Compton scattering in radiotherapy applications.

### Single and multiple elastic scattering

The screened Rutherford model is used to describe single elastic scattering. The PDF of the cosine of the elastic scattering polar angle, μ, is given by the normalized screened Rutherford cross section

20 $$p(\mu ,\eta )=\frac{2\eta (1+\eta )}{{(1+2\eta -\mu )}^{2}},$$

where η is the screening parameter in the Rutherford potential. The total macroscopic cross section for elastic scattering Σ_SR_ and screening parameter η are calculated on the fly using equations (4.7.6–4.7.9) in EGSnrc manual^16^.

The cumulative effect of a large number of elastic scatterings over a given path length is described by a multiple scattering distribution. The theory of Goudsmit and Saunderson^49^ (GS) provides an exact solution to the multiple scattering problem if the single scattering cross section is constant over the step. Using the screened Rutherford model to represent the single scattering, we arrive at the distribution of μ over a given path length s

$${F}_{GS}(\mu ,t,\eta )=\mathop{\sum }\limits_{l=0}^{\infty }\left(l+\frac{1}{2}\right){P}_{l}(\mu )\exp [-t{g}_{l}(\eta )],$$

$${\rm{t}}={\varSigma }_{SR}s,$$

21 $${g}_{l}(\eta )={\int }_{-1}^{1}[1-{P}_{l}(\mu )]p(\mu ,\eta )d\mu ,$$

where P_*l*_ is the *l* ^th^ Legendre polynomial and g_*l*_ is the GS-moments of single elastic scattering distribution. Equation (21) gives the exact form of GS-moments g_*l*_ but the integration is extremely time consuming especially for high order terms. Therefore, for the 100^th^ or higher order g_*l*_, the small-angle moments given by Kawrakow and Bielajew^50^ are used instead

$${g}_{SA,l}(\eta )=1-y{K}_{1}(y),$$

22 $$y=2\sqrt{l(1+l)\eta },$$

where K_1_ is the first order modified Bessel function of the second kind. According to our calculation, for η within the interval [1e-9, 1e-3], the relative differences between GS-moments and small-angle moments after the first 100 terms are less than 0.01%. Thus, small-angle moments provide a good approximation for high order g_*l*_.

The F_GS_ table cannot be used directly in MC simulation to sample multiple scattering because the steep distribution of F_GS_ requires an unrealistic amount of memory space to ensure accuracy of the linear interpolation. Kawrakow and Bielajew solved the problem by employing a variable transformation to make the distribution flat and easy for numerical representation^50^. The formula of the variable transformation is

23 $$u=(1+A)\left(1-\frac{2A}{1+2A-\mu }\right),$$

where A is introduced as a “spreading” parameter for the screened Rutherford distribution. Thus, we have the distribution of the new variable $u$ expressed as

24 $${q}_{SR}(u,t,\eta )=\frac{2A(1+A)}{{(1+A-u)}^{2}}{F}_{GS}\left(1-\frac{2Au}{1+A-u},t,\eta \right).$$

The free parameter A can be selected arbitrarily to make the distribution of q_GS_ as close to unity as possible, which requires that

25 $$\frac{\partial }{\partial A}{\int }_{0}^{1}{[{q}_{SR}(u,t,\eta ,A)-1]}^{2}du=0.$$

The above integro-differential equation is solved numerically to obtain A for each combination of t and η. These numerical solutions are used to fit a semi-empirical expression of A(t, η). The result

26 $$A(t,\eta )=\eta (t+4)(-1.293+x[1.434-x(0.07152-0.002553x)]),$$

with x = ln(t), is hard-coded in the MC program for efficient evaluation of A on-the-fly.

Based on Eq. (24), a three-dimensional inverse q_SR_ table (i.e. u values) as a function of ξ, t, and η is pre-compiled using MATLAB following a similar treatment to the previous sections. The actual number of GS series used to ensure the convergence of F_GS_ varies between ~10^2^ and 10^4^, depending on the values of t and η. F_GS_ is valid for any scattering angle, so cosine μ falls in [−1, 1] and u in [0, 1]. The range of t and η are set as [1e1, 1e5] and [1e-9, 1e-3] respectively. They are wide enough to cover the typical values of t and η associated with energies and materials seen in radiotherapy applications. The sampling of multiple scattering can thus be fulfilled by the interpolation of u from the inverse q_SR_ table and a variable change based on Eq. (23) to deliver μ.

GS theory requires that the elastic scattering cross section remains constant over the step. However, electrons lose energy continuously as they travel in matter. Since the elastic scattering cross section is a strong function of electron energy, it can change significantly in a large step. To account for energy loss, electron energy at the middle of the step is taken as the equivalent electron energy over the step, namely

27 $$\tilde{T}=T-\frac{s}{2}L\left[T-L(T)\frac{s}{2}\right],$$

where T is the initial kinetic energy of the electron, s is the path length, and L is restricted stopping power. The equivalent electron energy T̃ is used in the subsequent calculation of elastic total cross section Σ_SR_ and screening parameter η.

### Electron step and boundary crossing algorithm

The actual trajectory of a condensed electron step is not a straight line. Following PENELOPE^51^, the random-hinge method is adopted to correct longitudinal displacement and lateral deflection of the electron step. This method can be described as follows: (1) A hinge is randomly selected on the initial linear path. (2) Move the electron to the hinge and change its direction based on multiple scattering theory. (3) Move the rest of the path along the new direction to arrive at the final position.

In condensed-history techniques, a large number of interactions are grouped in a single step to speed up the simulation of electron transport at the expense of accuracy. Therefore, electron step size should be selected appropriately to balance efficiency and accuracy. We put several restrictions on the electron step size. The first is a user-defined global step limit S_MAX_ with a default value of 5 cm. The second is a fractional energy-loss limit S_E_, defined as the path length corresponding to a user-supplied maximum fraction of energy loss, which is 0.25 by default. The third step size limit is the distance S_C_ to the next catastrophic interaction site, which is obtained from

28 $${S}_{C}=-\frac{\mathrm{ln}\,\xi }{{\varSigma }_{B}+{\varSigma }_{M}},$$

where ξ is a random number sampled uniformly between 0 and unity and Σ_B_ and Σ_M_ are the total-catastrophic Bremsstrahlung and Møller cross sections. To account for variations of catastrophic cross sections due to continuous energy loss, fictitious interaction is introduced following the description of Hissoiny^19^.

Another restriction on step size is related to the boundary crossing problem. We employ a slightly modified version of EGSnrc’s PRESTA boundary crossing algorithm^52^. In this algorithm, the perpendicular distance to the nearest boundary S_⊥_ and the skin depth S_k_ are calculated for each step. Skin depth corresponds to N_k_ elastic mean free paths (MFPs) where N_k_ is a user-defined integer. The default setting is 300. When S_⊥_ > S_k_, the electron is regarded as far away from the boundaries. Thus the next step is confined within the current volume and a random-hinge correction is applied. In this case, electron step size S is given as

29 $$S=min\{{S}_{MAX},{S}_{E},{S}_{C},{S}_{\perp }\}.$$

when S_⊥_ < S_k_, the electron is close to the boundary. The random-hinge correction is turned off and the electron is allowed to travel on a straight line to the boundary. Meanwhile, to avoid an electron that is leaving the boundary from taking a large step without any correction, skin depth restriction is applied. Given all this, the electron step size S, in this case, is taken as

30 $$S=min\{{S}_{MAX},{S}_{E},{S}_{C},{S}_{k},{t}_{seg}\},$$

where t_seg_ is the length of the line segment to the boundary.

### General geometry

A geometry modeling module is introduced to characterize the geometry of our MC dose engine for particle tracking^31^. A complex three-dimensional heterogeneous geometrical system can be defined through a simple and variable input format. To model a system, a combination of boundary representation and constructive-solid-geometry techniques is used. The geometrical system is described in two steps. In the first step, zone-primitives using the boundary representation are defined. Then, the modeled system is constructed by creating a hierarchy of primitives through embedding. In the geometry module, zone primitives are the building blocks. The first- and second-order surfaces (e.g., planes, cylinders, cones etc.) in Cartesian space are used to describe the boundaries of zone primitives. A surface is defined by specifying the coefficients of the following quadratic function^31^

31 $$F(x,y,z)={Q}_{x1}{(x-{x}_{0})}^{2}+{Q}_{x2}(x-{x}_{0})+{Q}_{y1}(y-{y}_{0})+{Q}_{z1}{(z-{z}_{0})}^{2}+{Q}_{z2}(z-z)+{Q}_{r}.$$

A surface is defined by its type, the parameters specific to the surface type, and the orientation of the surface relative to the coordinate axes in the input file. Two essential functions, independent of the physics of the particle transport and sampling schemes, are provided by the geometry package for particle tracking. First, a zone index is returned through a given spatial position within the system. This is helpful in the identification of the composition and density of the zone. Second, by knowing the position, zone index, and direction of the transported particle, the geometry package provides the distance to the zone boundary in a specified direction. The combination of these two functions with appropriate sampling schemes and physics is used to track the transported particle through the medium.

## Results

To evaluate the accuracy of PTM, photons and electrons with various energies in both homogeneous and multilayered phantoms were simulated and compared with EGSnrc/DOSXYZnrc^53^ (EGSnrc hereafter) simulations. EGSnrc was selected as a benchmark because it is widely used in radiotherapy applications and has been extensively validated against a variety of experimental measurements. The built-in PEGS4 data file 700ICRU was used for EGSnrc simulation. Electron and photon cutoff energies were set to 0.7 and 0.01 MeV, respectively. The Bethe-Heitler cross sections were used for Bremsstrahlung and pair production with the angular sampling method set to simple. Bound Compton scattering and Rayleigh scattering were turned on. Spin effects were turned off for the benchmark studies but the influence on dose distributions was evaluated. The other EGSnrc parameters were set to default. PTM simulations were performed in a batched fashion. The total number of histories was evenly divided into 10 batches. For each batch, dose deposition to each voxel was recorded to perform statistical analysis.

To further test the performance of PTM and to demonstrate its function as a general-purpose MC transport code, we performed simulations of two realistic situations and compared dose distributions with measurements. In the first case, a cobalt-60 teletherapy treatment head was modeled including a Co-60 capsule source, lead shield, iron shell, a fixed primary collimator, and a group of adjustable secondary collimators. The dimensions of these geometries followed the description of the manufacturer. Different field sizes were simulated by adjusting the position and inclination of the secondary collimators. In the second case, a phase space file was used to simulate a 9 MeV electron beam. Percent depth dose (PDD) curves and lateral profiles were compared with measurements. The experimental measurements for a Co-60 therapeutic unit (THERATRON PHOENIX) were performed at the Institute of Nuclear Medicine, Oncology and Radiotherapy (INOR), Abbottabad, Pakistan using a Farmer-type ionization chamber (IBA-FC65-G) connected to a PTW UNIDOS E, attached to an IBA blue water phantom. PDD curve for the 9 MeV electron beam was measured using an IBA CC13 ion chamber (300 V bias) and an IBA reference diode RFD 3843 (no bias) in an IBA blue water phantom controlled with an IBA OmniPro-Accept v6.4 software for the applicator of 15 × 15 cm^2^ at 100 cm source-to-surface distance (SSD). The corresponding electron beam profiles were measured using an IBA electron diode EFD 4011 (no bias) and the same IBA reference diode RFD 3843 (no bias) at various depths in water at 100 cm SSD. In both PDD and profile measurements, step-by-step mode, medium detector sensitivity and 1000 ms measurement time were selected in the OmniPro-Accept. The IBA CC13 ion chamber has an active volume of 0.13 cm^3^ and inner radius of 3 mm. Both the wall and the central electrode are made of air equivalent plastic C-552. Hence, the effective point of measurement of 1.5 mm was used for CC13. For electron diode EFD 4011, the effective point of measurement of 0.5 mm was used. After beam scans, we converted the electron ionization values into the PDD values using pre-defined TG-51 protocol included in the OmniPro-Accept software, which uses stopping-power ratios for realistic electron beams instead of mono-energetic stopping-power ratios. The measurement uncertainty was estimated as less than 2% considering Ptp < 0.1%, Pelec < 0.1%, Pion < 0.1%, Ppol < 0.4%, Pgr < 0.2%, and detector positioning uncertainty of 0.5%.

### Water phantom

**Figure 1:**
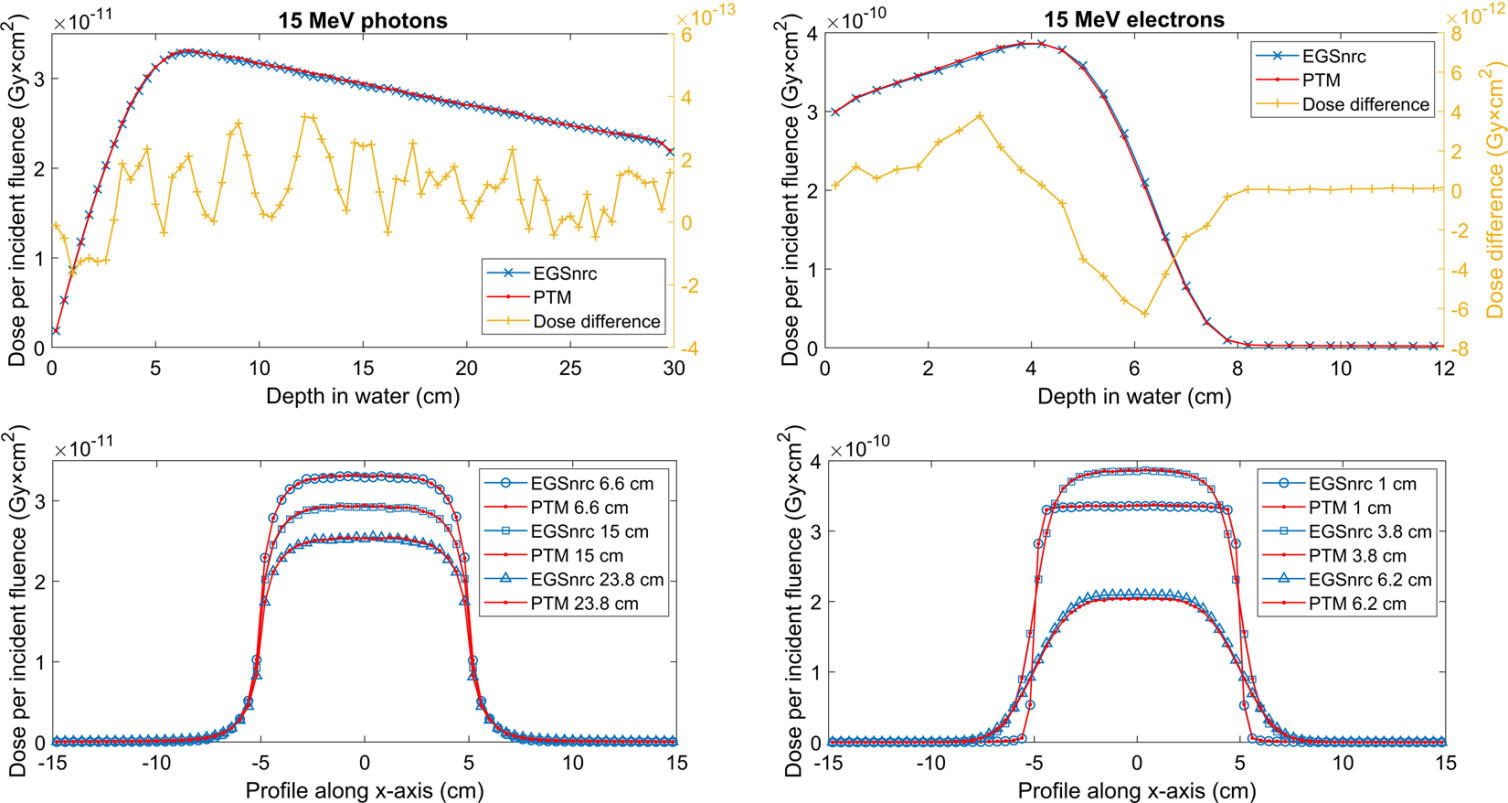
Depth dose curves (top row) and lateral profiles at different depths (bottom row) of the 15 MeV photon (left column) and electron (right column) 10 × 10 cm^2^ parallel beams in a 30×30×30 cm^3^ water phantom. PTM results are shown in red lines while EGSnrc results are shown in blue lines. Absolute differences of depth doses are shown in yellow lines (top row) with y-axis on the right. The same color-style applies for all the other figures. The standard deviations of MC simulations (<0.2%) are too small to be visible, hence not shown in this and all the other figures.

**Figure 2:**
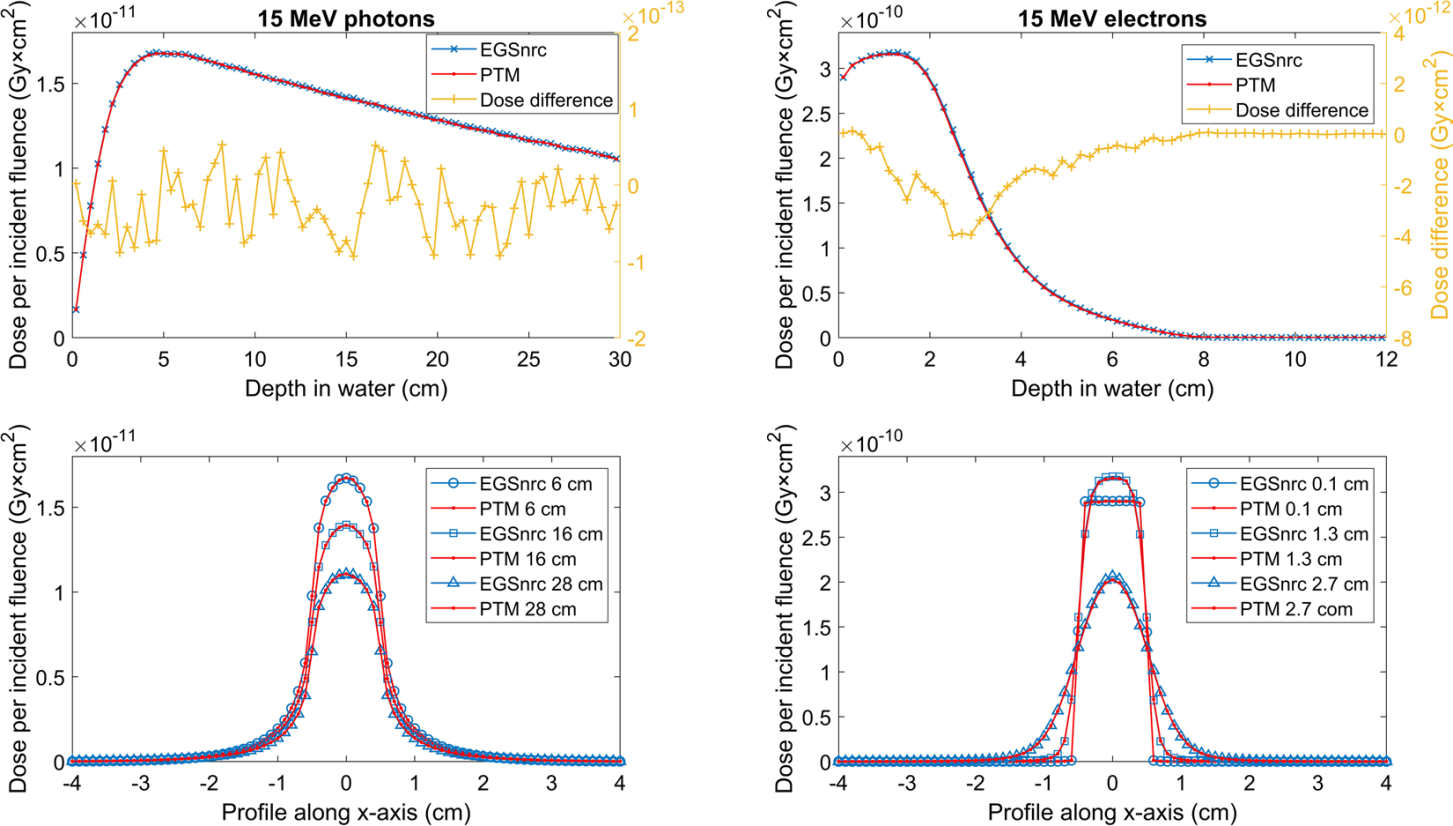
Depth dose curves (top row) and lateral profiles at different depths (bottom row) of the 15 MeV photon (left column) and electron (right column) 1 × 1 cm^2^ small parallel beams in a 30 × 30 × 30 cm^3^ water phantom.

We first investigated the performance of PTM on a homogeneous water phantom. The phantom consists of 75 × 75 × 75 cubic voxels with a voxel size of 0.4 × 0.4 × 0.4 cm^3^, which was deemed to be sufficient for a benchmark study. Parallel beams with a field size of 10 × 10 cm^2^ were used. Particles impinged on the top surface of the phantom along the z-axis. A total number of 1 billion and 100 million histories were generated for the photon and electron beam, respectively, to achieve a standard deviation on the order of 0.2% of the maximum dose. The depth dose and lateral profiles at different depths for a 15 MeV monoenergetic photon and electron beam are presented in Fig. 1. The depth dose is on the central axis along depth direction. To compare with EGSnrc, data shown in Fig. 1 are absolute doses normalized by particle fluence. Error bars are not displayed since they are usually smaller than the symbols. PTM simulations agreed well with EGSnrc with the largest dose discrepancy of 6 × 10^−12^ Gy × cm^2^ (3% of local dose) for electron beam observed in the dose falloff region. Gamma indices of the three-dimensional dose distributions were calculated using a 2 mm and 2% acceptance criteria^54^. All significant voxels (those having a dose higher than 20% of the dose maximum) passed the test with an average gamma index of 0.104 for the photon beam and 0.136 for the electron beam. Gamma test results are summarized in Table 1. Similar setups were used to simulate 1 × 1 cm^2^ small beams but with a voxel size of 0.1 × 0.1 × 0.4 cm^3^ for photon beam and 0.1 × 0.1 × 0.2 cm^3^ for electron beam. As shown in Fig. 2, PTM simulations agreed well with EGSnrc with the largest dose discrepancy of 4 × 10^−12^ Gy × cm^2^ (2% of local dose) for electron beam observed in the dose falloff region. Electron spin effects on the simulated dose distributions were studied by turning on the spin option in EGSnrc. A summary of gamma indices for 15 MeV photons and electrons impinging on water with spin on is shown in Table 2.

**Table 1:** Summary of gamma indices for various simulation scenarios.

Particle	Beam	Phantom	Total voxel	Significant voxel	γ_max_	γ̅_sv_	γ_sv_ < 1
**15 MeV photons**	10×10 cm^2^, parallel	water	421875	51466	0.7055	0.104	100%
**15 MeV electrons**	10×10 cm^2^, parallel	water	421875	12233	0.6587	0.1362	100%
**15 MeV photons**	10×10 cm^2^, parallel	water-lung-water	421875	53848	0.9319	0.2002	100%
**15 MeV electrons**	10×10 cm^2^, parallel	water-lung-water	421875	19415	0.5458	0.1212	100%
**15 MeV photons**	10×10 cm^2^, parallel	tissue-bone-tissue	421875	50796	0.8003	0.1788	100%
**15 MeV electrons**	10×10 cm^2^, parallel	tissue-bone-tissue	421875	9145	0.6196	0.1557	100%
**Co-60 γ-rays**	5×5 cm^2^, SSD 80 cm	water	60	43	0.6797	0.1072	100%
**Co-60 γ-rays**	10×10 cm^2^, SSD 80 cm	water	60	48	0.703	0.223	100%
**Co-60 γ-rays**	20×20 cm^2^, SSD 80 cm	water	60	54	0.8374	0.1939	100%
**9 MeV electrons**	15×15 cm^2^, SSD 100 cm	water	951	615	1.112	0.329	99.84%

**Table 2:** Summary of gamma indices for 15 MeV beam in a water phantom with spin effects turned on.

Particle	Total voxel	Significant voxel	γ_max_	γ̅_sv_	γ_sv_ < 1
**Photons**	421875	51435	0.7594	0.1335	100%
**Electrons**	421875	12414	1.1245	0.3267	99.69%

**Figure 3:**
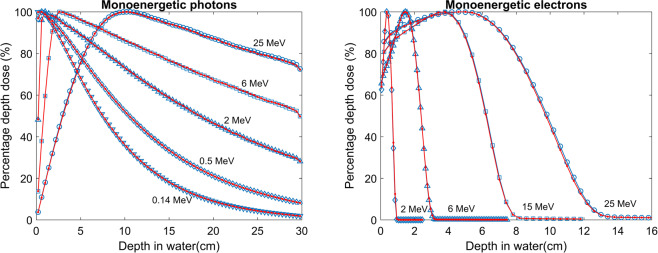
PDD of the 25, 6, 2, 0.5 and 0.14 MeV monoenergetic photon beams (left) and the 25, 15, 6 and 2 MeV monoenergetic electron beams (right) in a water phantom. PTM results are shown in red lines with dots while EGSnrc results are shown in blue lines with open symbols.

A water phantom with 15 × 15 × 75 voxels was used to evaluate the performance of PTM at various particle energies. Voxel size was 2 × 2 × 0.4 cm^3^ for all the photons and high energy electrons (15 and 25 MeV) while a smaller voxel of 2 × 2 × 0.1 cm^3^ was used for low energy electrons (2 and 6 MeV) due to the limited range. All beams were modeled as a 6×6 cm^2^ parallel beam. A total number of 10 million photons and 1 million electrons were simulated. Calculated voxel doses were normalized with respect to the maximum dose in the phantom. The same D_max_ was used to normalize the two datasets. PDD curves for 25, 6, 2, 0.5, and 0.14 MeV monoenergetic photon beams and 25, 15, 6, and 2 MeV monoenergetic electron beams are presented in Fig. 3. Dose differences were less than 2% for all the voxels except for those in the fall-off area of the 2 MeV electrons, where dose differences of about 3% were observed.

### Slab geometries

**Figure 4:**
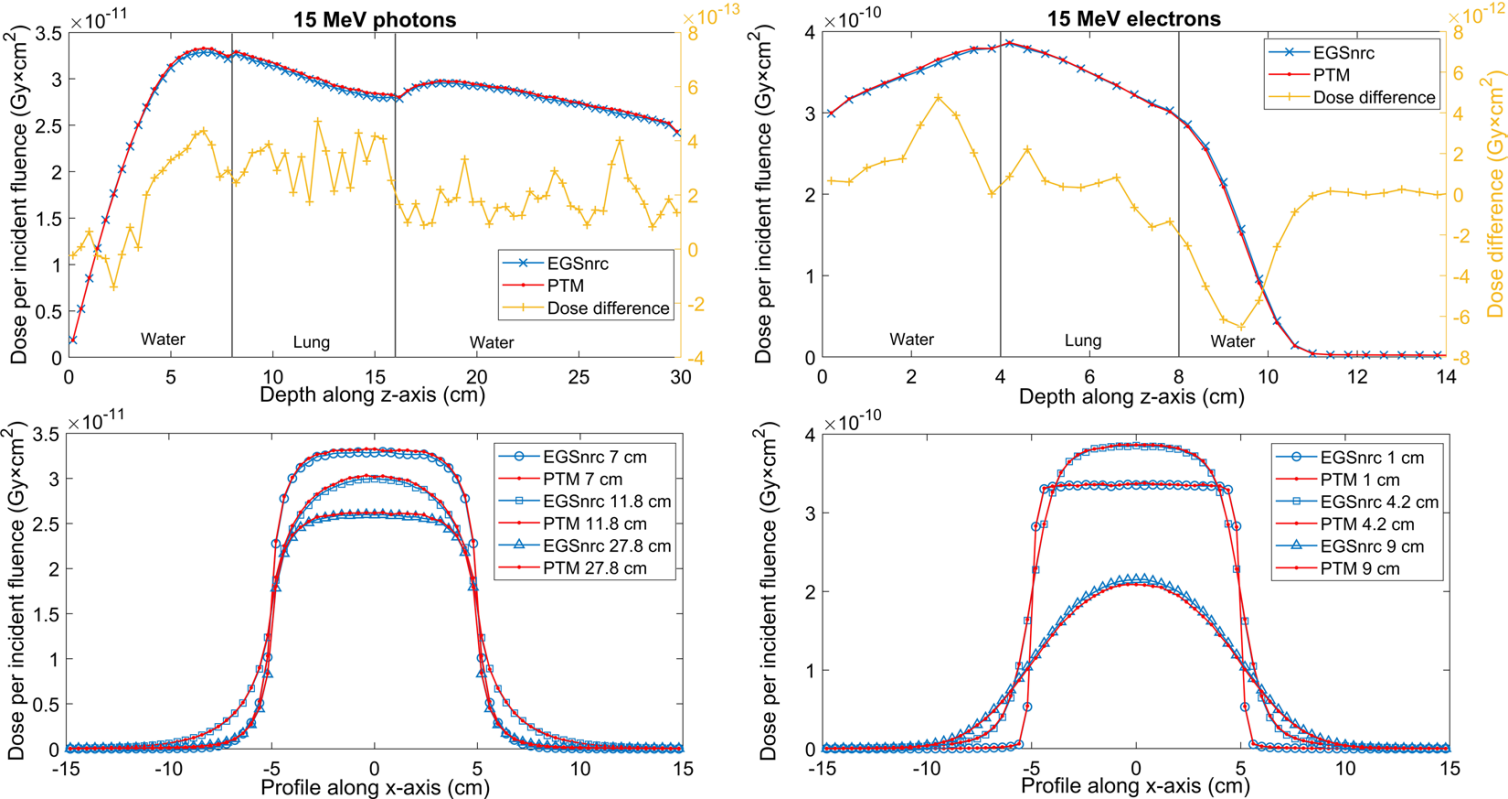
Depth dose curves (top row) and lateral profiles at different depths (bottom row) of the 15 MeV photon beam on an 8-8-14 cm, water-lung-water multilayered phantom (left column) and electron beam in a 4-4-22 cm, water-lung-water multilayered phantom (right column).

**Figure 5:**
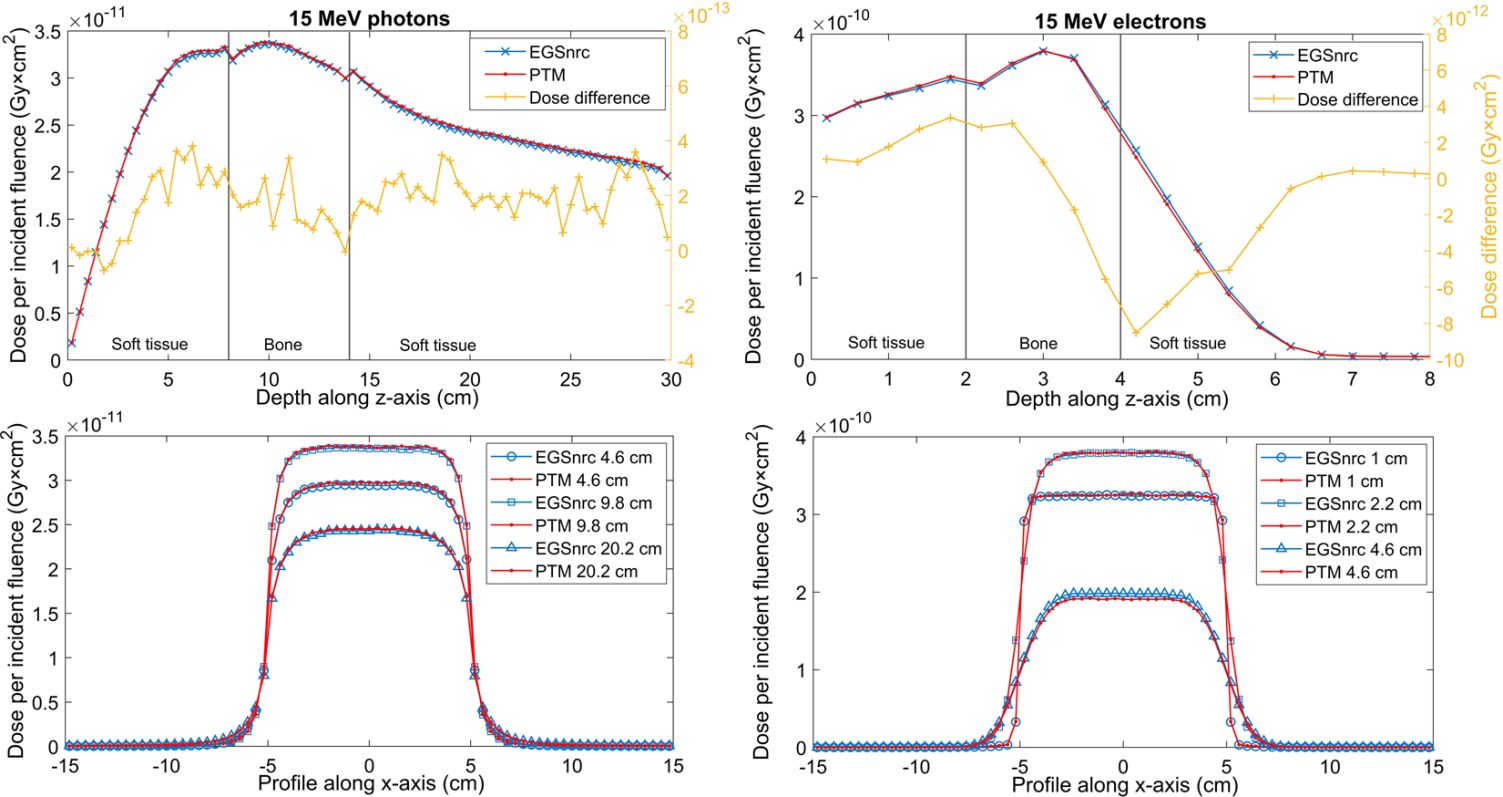
Depth dose curves (top row) and lateral profiles at different depths (bottom row) of the 15 MeV photon parallel beam on an 8-6-16 cm, tissue-bone-tissue multilayered phantom (left column) and electron parallel beam in a 2-2-26 cm, tissue-bone-tissue multilayered phantom (right column).

**Figure 6:**
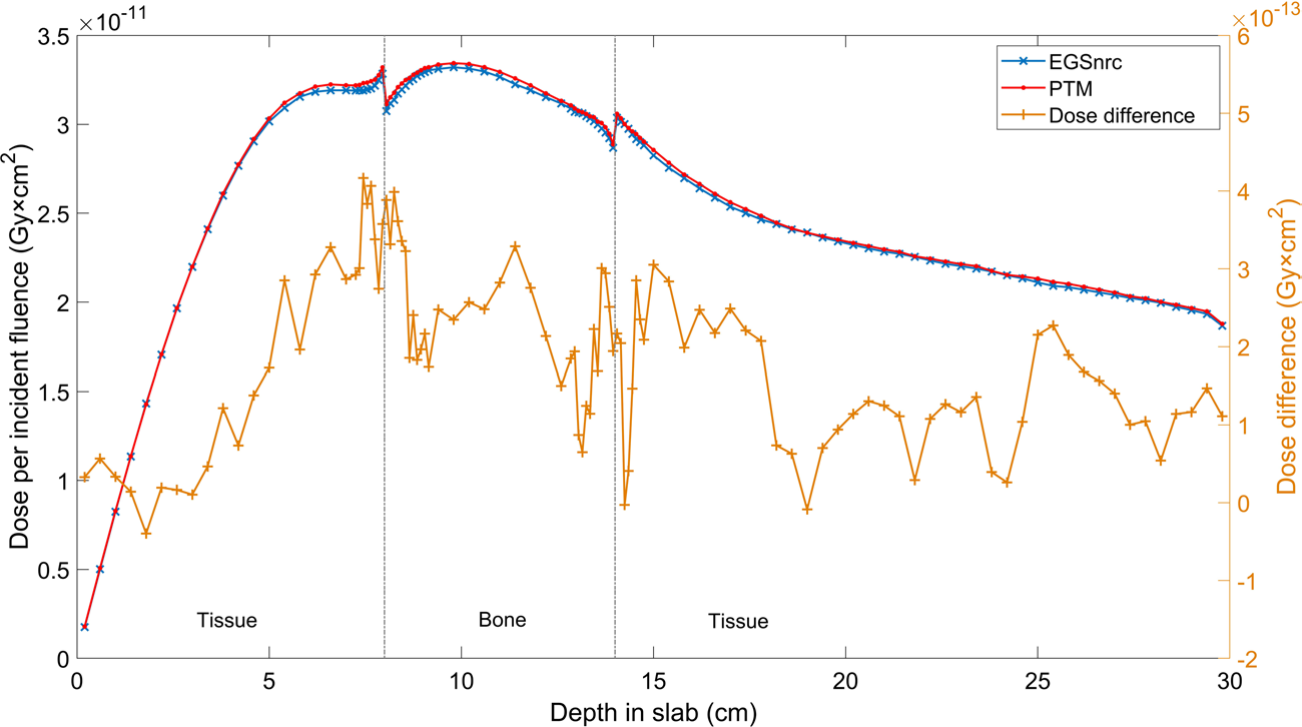
Depth dose curves of a parallel 15 MeV photon beam on an 8-6-16 cm, tissue-bone-tissue multilayered phantom with a voxel size of 0.4 × 0.4 × 0.1 cm^3^ near the interfaces.

**Figure 7:**
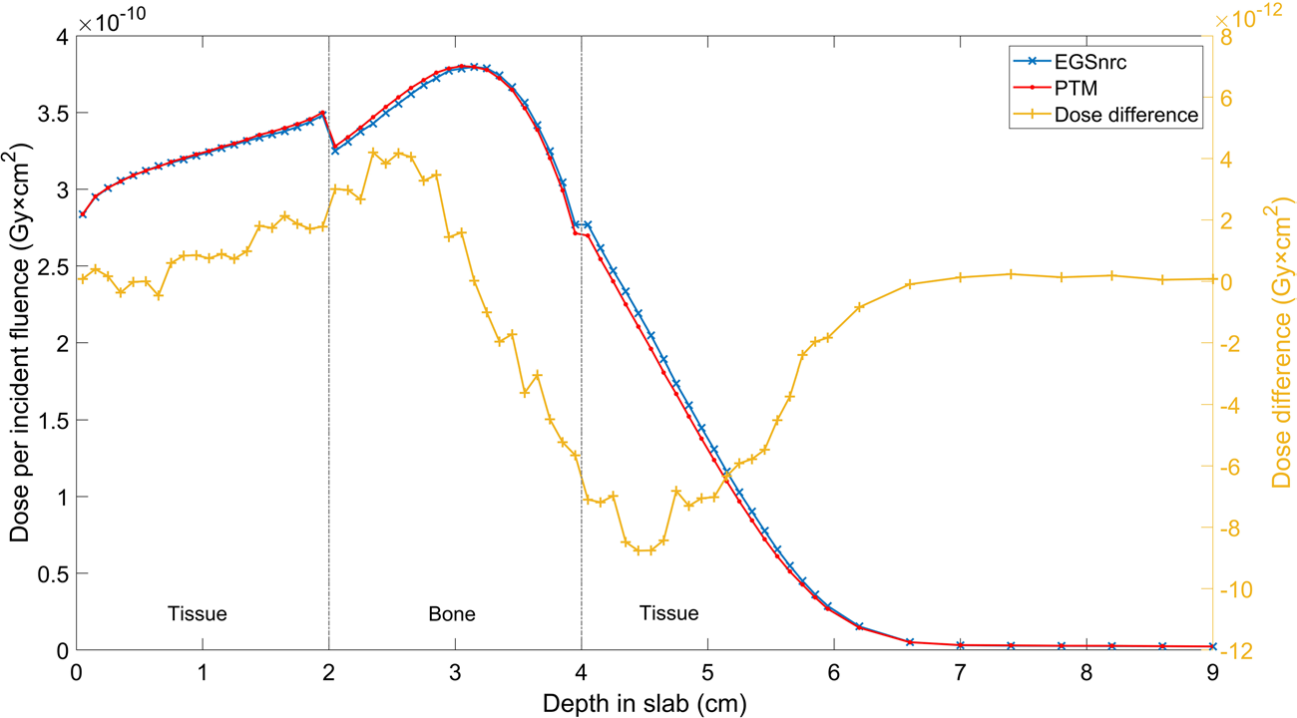
Depth dose curves of a parallel 15 MeV electron beam on a 2-2-26 cm, tissue-bone-tissue multilayered phantom with a voxel size of 0.4 × 0.4 × 0.1 cm^3^ near the interfaces.

Multilayered slab geometries were designed to test the performance of PTM for various materials and, most importantly, under electron non-equilibrium conditions. The same phantom of 75 × 75 × 75 voxels with a voxel size of 0.4 × 0.4 × 0.4 cm^3^ was employed. The first slab geometry was water-lung-water with a thickness configuration of 8-8-14 cm for the photon beam and 4-4-22 cm for the electron beam. The second slab geometry was tissue-bone-tissue with a thickness of 8-6-16 cm for the photon beam and 2-2-26 cm for the electron beam. Again, 10 × 10 cm^2^ parallel beams were used. Overall, 1 billion photons and 100 million electrons were generated to achieve good statistics. The depth dose and lateral profiles at different depths for a 15 MeV monoenergetic photon and electron beam impinging on the slab phantoms are compared between PTM and EGSnrc in Figs. 4 and 5. The standard deviations of Monte Carlo simulations were smaller than symbols and thus not shown. In general, good agreement between the two was found near the slab interfaces. In Fig. 4, the PTM result was 1.3% higher than that of EGSnrc upstream of the water-lung interface and was less than 1% (local dose) near the interface for the 15 MeV photon beam. While in Fig. 5, the bone-tissue interface in the falloff region saw the largest dose difference of 7 × 10^−12^ Gy × cm^2^ (3% of local dose) for the 15 MeV electron beam. Gamma index statistics were presented in Table 1. All significant voxels met the 2%/2 mm criteria and the average gamma indices were between 0.12 and 0.2. To investigate the implementation of multiple scattering and Mott corrections at the interfaces between different tissues, we repeated the tissue-bone-tissue slab simulations using 1 mm voxels near the interfaces. Consistency was found between the 4 mm voxels (Fig. 5) and the 1 mm voxels for both 15 MeV photons (Fig. 6) and 15 MeV electrons (Fig. 7).

### Cobalt-60 machine

**Figure 8:**
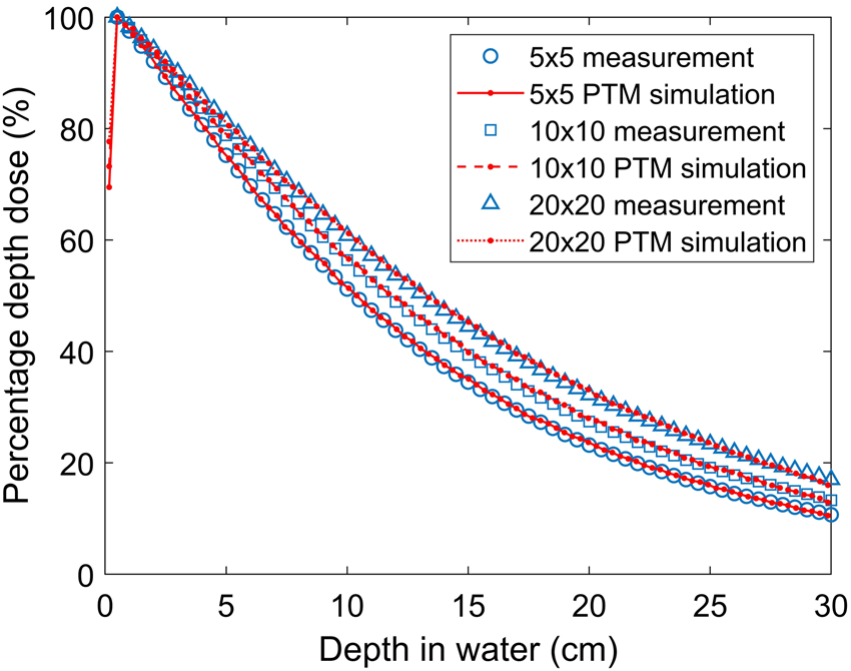
Simulated (lines) and measured (symbols) PDD along the central axis for a Co-60 teletherapy machine at a SSD of 80 cm with field sizes of 5 × 5 (solid line and circles), 10 × 10 (dashed line and squares) and 20 × 20 (dotted line and triangles) cm^2^ at the surface of the water phantom.

A cobalt-60 teletherapy treatment head was modeled based on the geometries detailed by the manufacturer. The Co-60 source was modeled as a cylinder with isotropic emission of 50% 1.17 MeV photons and 50% 1.33 MeV photons. The γ-rays emitted from the source were collimated by a fixed primary collimator and two pairs of jaw collimators to produce a rectangular beam of varying field sizes. A SSD of 80 cm was used for both simulations and measurements. A 30 × 30 × 30.03 cm^3^ water phantom with 1 × 1 × 0.33 cm^3^ voxels was employed in the simulation. The size of the voxel was selected to match the sensitive volume of the ionization chamber. Total history numbers were 1, 2, and 8 billion for respective field sizes of 5 × 5, 10 × 10, and 20 × 20 cm^2^ at the surface of the phantom. Measurements were normalized to the dose at a depth of 0.5 cm. Simulation results were normalized to the maximum dose, which is the dose of the second voxel along the central axis with a voxel center depth of 0.495 cm. PDD curves along the central axis for different field sizes obtained from PTM simulations were compared with measurements. Figure 8 shows the excellent agreement between PTM simulations and measurements with less than 3% of local dose (less than 1% of maximum dose). Gamma tests were performed for voxels along the central axis, and Gamma indices are summarized in Table 1.

### Electron linear accelerator

**Figure 9:**
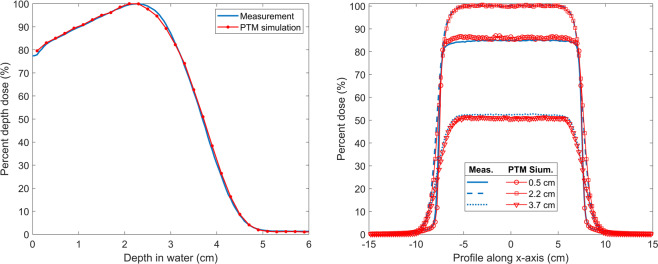
Simulated (red line with symbols) and measured (blue line) PDD along central axis (left) and profiles at the depth of 0.5, 2.2 and 3.7 cm (right) for a nominal 9 MeV electron beam with a field size of 15 × 15 cm^2^ at SSD = 100 cm on a Varian Clinac 2100 C/D machine.

The phase space file for a Varian Clinac 2100 C/D operating in the electron mode with a nominal electron beam energy of 9 MeV and a field size of 15  ×  15 cm^2^ at SSD = 100 cm was downloaded from the IAEA database^55^ (https://www-nds.iaea.org/phsp/electron1). The phase space file was tallied right below the second scraper at z = 78.5 cm. The third scraper at z = 95 cm was modeled in our simulation with an inner opening of 14.1 × 14.1 cm^2^ and a thickness of 1.4 cm. The material of the third scraper was steel. A water phantom consisting of 149 × 149 × 50 voxels with a voxel size of 0.2 × 0.2 × 0.2 cm^3^ was used. The voxel size was selected to match the sensitive volume of the electron diode detector. A total of 1 billion histories were generated to achieve a standard deviation of about 0.3% of the maximum dose. Comparisons with experimental measurements for PDD and lateral profiles are presented in Fig. 9 where PTM accurately reproduced the measurements in the buildup and falloff regions, at the depth of maximum dose, and along the bremsstrahlung tail of the electron beam. The summary of gamma indices can be found in Table 1.

### Fano test

**Figure 10:**
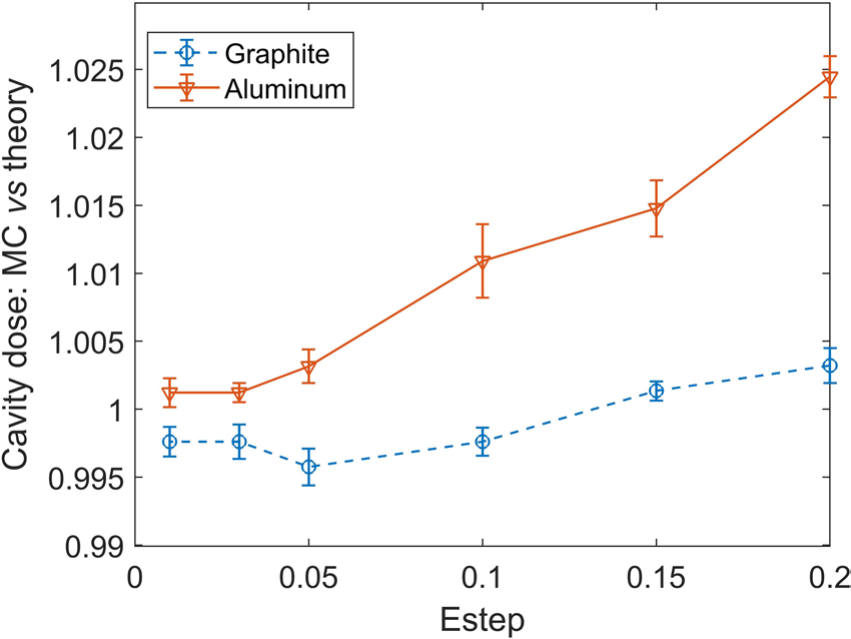
The ratio of the simulated and theoretical Fano Cavity dose as a function of electron fractional energy loss step size for graphite (dashed line and circles) and aluminum (solid line and triangles).

Fano Cavity test was performed to evaluate the difference between simulated and theoretical ion chamber response as a function of step size. The thickness of the wall and cavity was 4 mm and 2 mm, respectively. The diameters of the ion chamber and the cavity were 3 cm and 2 cm. Incident beam was 1.25 MeV monoenergetic photon beam perpendicular to the flat side of the chamber. Theoretical ion chamber dose was estimated to be 5.3528 × 10^−12^ Gy×cm^2^ and 5.1637 × 10^−12^ Gy×cm^2^ for graphite and aluminum using equations in the reference paper^56^ and EPDL 2017 data library^30^. To satisfy the idealized conditions of theoretical calculation, we applied the following simulation setup. The cavity had the same material as the wall but with much lower density. Scattered photons were removed and replaced with the original 1.25 MeV photons to compensate wall attenuation. Bremsstrahlung radiation was disabled to ensure that energy transfer is equal to energy absorption. Density correction for the calculation of restricted stopping power was ignored. Cutoff energy for photons and electrons were set as 10 keV and 1 keV, respectively. Photon cross section was increased by a factor of 100 to improve simulation efficiency. Other parameters were kept as default. The results for graphite and aluminum ion chamber responses are presented in Fig. 10. The differences between simulation and theory converged to about 0.2% at very small steps, indicating the accuracy of the electron transport algorithm in PTM. A fractional energy loss step size of 0.1 or smaller for electrons was necessary to keep the ion chamber response difference within 1%.

## Discussion

Most MC particle transport platforms habitually adopt the acceptance-rejection method to generate observations from a distribution^16,33,41,45,51,57^. There are several advantages to rejection sampling in MC simulation. It can be easily implemented to sample a variable from an arbitrary PDF. If the PDF is well represented by some theoretical models, then no additional memory space is needed to store the numerical representation of the PDF. This is true in most cases since very accurate theoretical models have been developed for most of the physical processes in particle transport problems. However, problem arises when one attempts to adapt the algorithm on a single instruction multiple data (SIMD) architecture platform, such as GPU. State-of-the-art GPUs have dozens of cores and tens of thousands of threads. Since each particle interaction with the medium is independent by nature, it is possible to take advantage of GPUs to simulate a large number of histories in parallel to accelerate MC simulation. However, SIMD architecture can only implement a single instruction in a warp (a group of 32 threads) at a time. If some threads accept the sampling while the other threads reject the sampling, those threads that have already accepted values must wait until all the other threads in the warp get an accepted value. Then all the threads in the warp will move together to the next instruction. This is known as thread divergence, which may pose a significant efficiency-penalty for programs running on SIMD architecture. To circumvent difficulties one will encounter in the future implementation of this code on GPU, we employed an inverse transform sampling technique throughout the current MC code following the treatment of Jia^20^. In this method, the CDF of a variable ξ = F(x) is first calculated based on its PDF. Then an inverse transformation is performed to obtain the inverse function of the CDF, x = F^–1^(ξ). During the simulation, this algorithm needs only to pick a random number between 0 and 1 once to represent the probability ξ and calculate the value of x using the inverse function. This avoids the thread divergence entirely.

**Figure 11:**
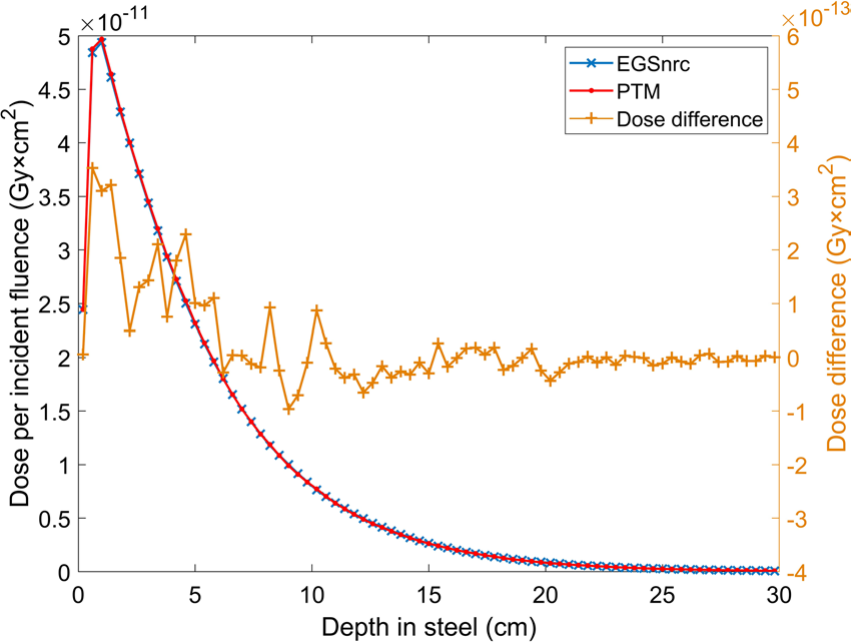
Depth dose (left y-axis) and dose difference (right y-axis) of a parallel 15 MeV photon beam in a 30 × 30 × 30 cm^3^ steel phantom with a voxel size of 4 × 4 × 4 mm^3^.

**Figure 12:**
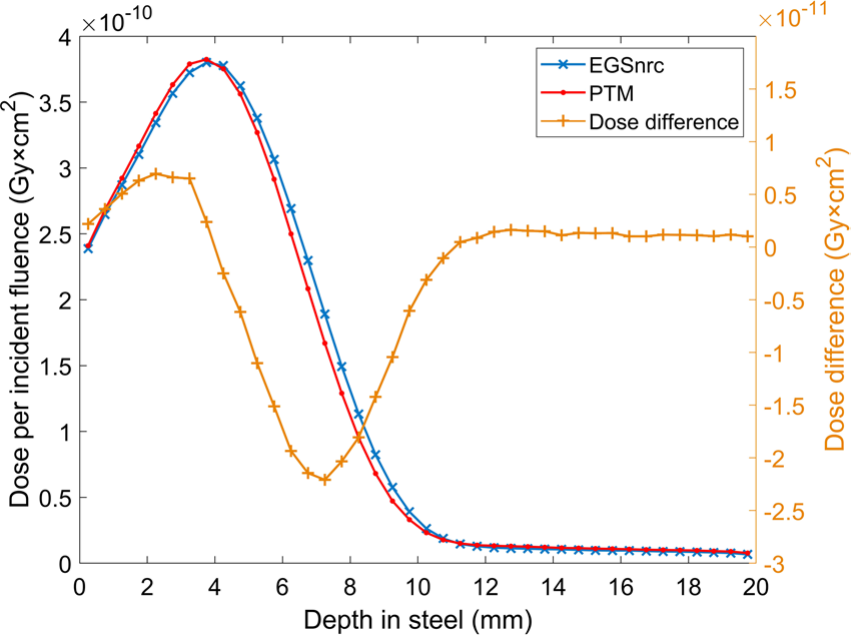
Depth dose (left y-axis) and dose difference (right y-axis) of a parallel 15 MeV electron beam in a 30 × 30 × 2 cm^3^ steel phantom with a voxel size of 4 × 4 × 0.5 mm^3^.

The primary goal of this CPU-based MC code is to validate the physical models and transport algorithms, as well as the inverse transform sampling method that has been implemented. Therefore, the accuracy of PTM is the first and foremost concern. We benchmarked PTM against EGSnrc using the DOSXYZnrc toolkit. EGSnrc is widely recognized in the radiotherapy community as a very accurate MC platform that has been demonstrated to allow an artifact-free simulation of the most stringent ion chamber response and backscattering problems^56^. Comparison with EGSnrc was first performed in a homogeneous water phantom for both photons and electrons at various energies. Absolute radiation dose as a function of depth and off-axis distance at three depths were directly compared with EGSnrc result without normalization to exclude the existence of any systematic error in PTM. As can be seen in Figs. 1 and 2, in most cases, the two curves that represent our result and EGSnrc result perfectly overlay. Figure 3 shows the excellent agreement between PTM simulation and that of EGSnrc for PDD curves of photons and electrons with different energies. Especially, the build-up region for photons and electrons and the Bremsstrahlung tail for electrons were well reproduced. A photon beam with energy as low as 140 keV was used in the benchmark study to justify the application of PTM in kV imaging dose calculations, such as kV CBCT and mammography. For the water phantom, the differences between PTM and EGSnrc were usually less than 1% of the maximum dose, except for the dose fall-off region of the 2 MeV electrons. In this high-gradient region, the differences were as large as 3-4% but the distance-to-agreement was in the sub-millimeter range. Thus, the PTM simulation result was still quite good for low energy electrons in a measure of the gamma index. Depth doses for photons and electrons in high Z material (steel) are shown in Figs. 11 and 12. The result of photon beam agreed well with less than 1% local dose discrepancy. Up to 10% local dose difference was observed in the dose falloff of the electron beam. This indicates that simplification of Bremsstrahlung cross section as a linear function may lead to inaccurate dose calculation for electron beam in high Z materials.

Another set of comparisons was carried out in two multilayered phantoms to test the performance of PTM in heterogeneous media where electron non-equilibrium is present. The two slab phantoms were a lung layer in water and a bone layer in soft tissue, as indicated in Figs. 4–7. Overall good agreement with EGSnrc was found for both photons and electrons, demonstrating the consistent performance of PTM in homogeneous and heterogeneous media. The differences were on the order of 1% of the maximum dose in most of the voxels, including those near the slab interfaces. This proves that the electron step algorithm and boundary crossing algorithm applied in PTM are very accurate. In the fast fall-off region of electron depth dose curves, differences of up to 2% were found but the distance-to-agreement were very small. The gamma test was performed to quantitatively evaluate the accuracy of PTM with EGSnrc as a benchmark. Table 1 summarizes the gamma index statistics for the six different combinations of beams and phantoms. Not a single voxel out of the 0.2 million significant voxels failed the gamma test under the 2%/2 mm acceptance criteria. The average gamma indices fell in the interval of [0.1,0.2] for all the six simulation scenarios.

Experimental measurement is the gold standard of benchmark studies. PDD curves in a water phantom were measured for a Co-60 teletherapy machine with field sizes of 5 × 5, 10 × 10 and 20 × 20 cm^2^ at a SSD of 80 cm. To reproduce the experiment in silico, the treatment head was modeled as detailed by the manufacturer and the secondary collimators were set following experimental setup. Figure 8 shows the comparison of PDD from PTM simulation and measurement for the three field sizes. PDD curves along the central axis were higher for larger field sizes due to the contribution of more scattering. Simulation agreed well with measurement with the difference being less than 1% of the maximum dose. A build-up region was found in the simulation but not in the measurement since the shallowest depth measured lay just below the build-up region. All significant voxels along the central axis met the 2%/2 mm gamma criteria, as shown in Table 1. The average gamma indices were below 0.23 for the three field sizes. This result shows that PTM is able to simulate a complex treatment head with satisfactory accuracy, encouraging its application in the clinical study.

A phase space file was used in the simulation of the dose distribution for a 9 MeV electron beam produced by a Varian Clinac 2100 C/D. Simulated PDD and profiles were in good agreement with measurements, as shown in Fig. 9. Differences on the order of 1.5% were found in the fall-off region of the PDD curve which might be due to the inaccuracy of the screened Rutherford cross section and the linear approximation of the Bremsstrahlung energy-weighted spectrum. The entrance doses and the lateral profiles of the electron beam were well reproduced. The gamma test passing rate for the significant voxels was as high as 99.84% under the 2%/2 mm criteria. Overall, the various benchmarks performed so far demonstrate the accuracy and reliability of PTM as a general-purpose MC code devoted to radiation therapy applications.

The electron step correction algorithm and the boundary crossing algorithm play a crucial role in the 3D dose distribution. It is argued that the introduction of an imaginary step in electron transport in the condensed history scheme bring about inherent error that vanishes as step size tends to zero^58^. According to our test, if we turn off the random-hinge correction of the electron step and disable the skin depth option for boundary crossing, the differences between our code and EGSnrc reached 3% for a global maximum step size S_MAX_ of 0.5 cm and 2% for a S_MAX_ of 0.2 cm. In sharp contrast, if we turn on these options, the difference decreased to the order of 1% even for a S_MAX_ of 5 cm. This suggests that PTM has mostly removed the electron step size artifacts. The main reason, we believe, is the implementation of a strict boundary crossing algorithm in the voxelized phantom. At the beginning of each step, we first determine if the electron is close to a boundary or not. This is done by calculating the perpendicular distance to the nearest boundary in units of elastic MFP and comparing with a user-defined threshold skin depth. If the electron is not within the skin depth, the maximum allowed step size will be limited to the perpendicular distance. For a cubic voxel with a side length of 0.4 cm, one could estimate that the average perpendicular distance to the nearest surface of a random point inside the voxel is less than 0.1 cm. Therefore, for high energy electrons in a phantom with small voxels, boundary crossing algorithm imposes a strong restriction on the step size. For low energy electrons, the fractional energy loss will be the dominant restriction regarding the step size. For example, the restricted stopping power of a 0.5 MeV electron in water is about 2.1 MeV/cm. If the fractional energy loss limit is 0.25, then the maximum allowed step size for this electron will be 0.06 cm. With these various restrictions imposed on the electron step, we are confident in the adaptation of a large global step size limit to improve the efficiency of the electron transport in large geometries such as the surrounding air while maintaining high accuracy in the phantom where energy deposition will be registered.

The screened Rutherford cross sections described in this paper provide a good approximation of the electron elastic scattering. Yet, the more accurate cross sections for elastic scattering could be those derived from partial wave analysis of the Dirac equation in the spin-coupled Coulomb field of the atom. A general formula of the cross section is obtained by Mott^59^. Outright use of Mott cross sections would make it difficult to ensure interpolation accuracy. Simplification is made in EGSnrc to add a Mott correction term to the screen Rutherford cross section to account for the relativistic spin effects^16^. Consequently, an additional rejection loop using the Mott correction as a rejection function is needed for the sampling of scattering polar angle from the multiple scattering distribution obtained from the screened Rutherford model. Spin effects are not modeled in PTM because we wanted to develop an algorithm that is completely based on inverse transform techniques rather than rejection techniques. To validate the models that have been implemented in PTM, we benchmarked against EGSnrc in the spin off mode, although spin on is the default option in EGSnrc. It is suggested that one would get better agreement with measurement for electron dose distribution by taking into consideration the spin effects^60–62^. To evaluate the extent to which spin effects will influence the simulation result in a typical radiotherapy application, we repeated the EGSnrc simulations of 15 MeV photons and electrons in a water phantom with spin on. The gamma test was performed using the 2%/2 mm acceptance criteria. Results are presented in Table 2. For the photon beam, 100% of significant voxels passed the test with an average gamma index as low as 0.134, suggesting negligible influence for the photon beam. The spin effects are completely neutralized by the random distributions of energy and direction of the secondary electrons produced by the primary photons. For the electron beam, a noted increase of average gamma index to 0.367 was observed. Nevertheless, the overall gamma test passing rate of 99.7% was acceptable. Ignoring spin effects does not significantly deteriorate the performance of PTM.

In this study, the PTM simulation time was found 1.2–1.6 times that of DOSXYZnrc with the same level of statistic uncertainty. The longer simulation time may primarily result from the added functionality of PTM for particle transport in both a general geometry (e.g., linac head or radioactive source) and a voxelized geometry at the same time, while DOSXYZnrc is optimized for dose simulations in a voxelized geometry.

## Conclusion

In this work, we have successfully developed PTM, a general-purpose Monte Carlo package for coupled photon-electron transport simulation on CPU. PTM employs the inverse transform sampling method instead of the conventional acceptance-rejection method throughout the implementation of various physical models. The accuracy of PTM has been validated through a series of benchmark studies against both EGSnrc simulations and experimental measurements for different combinations of beams, phantoms, and geometries. This study establishes a CPU framework for future GPU implementation of a coupled photon-electron transport code based on inverse transform sampling.

## Data availability

The data in this study is available from the corresponding author upon request.
